# A comparison of injured patient and general population valuations of EQ-5D health states for New Zealand

**DOI:** 10.1186/1477-7525-12-21

**Published:** 2014-02-18

**Authors:** Ross Wilson, Paul Hansen, John Langley, Sarah Derrett

**Affiliations:** 1 Injury Prevention Research Unit, Department of Preventive & Social Medicine, University of Otago, Dunedin 9054, New Zealand; 2 Department of Economics, University of Otago, Dunedin 9054, New Zealand

**Keywords:** EQ-5D, Health state preferences, New Zealand, Injury, Visual analogue scale

## Abstract

**Background:**

A near-universal finding internationally is that patient valuations of their own health, represented using the EQ-5D system, are mostly higher than general population valuations of the same EQ-5D states. This paper investigates whether this result also applies to New Zealand. Despite the EQ-5D’s widespread use for cost-utility analysis in New Zealand, in particular by the Pharmaceutical Management Agency (PHARMAC) for health technology assessments, no previous studies comparing patient and general population valuations have included data for New Zealand.

**Methods:**

Valuations of 13 EQ-5D health states from a 1999 survey of the New Zealand general population (n = 396) are compared with injured New Zealanders’ (n = 2099) valuations of their own health (also represented on the EQ-5D) collected between 2007 and 2009 in the Prospective Outcomes of Injury Study. Which EQ-5D dimensions are most strongly associated with the population valuations is also investigated.

**Results:**

Injured population valuations are higher (better-rated health) than general population valuations for all 13 health states considered except 11111 (no problems on any EQ-5D dimension). This difference, which tends to be larger the ‘worse’ the state, is statistically significant at the 10% level for most of the states. State 11111 is rated significantly lower by the injured population than the general population. Pain/discomfort is more important in determining valuations for the general population than for injured people, whereas problems with self-care are more important for the injured population; anxiety/depression is important in both general and injured population valuations.

**Conclusions:**

Consistent with the international literature, injured people’s valuations of their own health are mostly higher than the general population’s hypothetical valuations of the same EQ-5D states for New Zealand. These differences are practically significant in the sense that they are larger than minimally important differences for the EQ-5D from the literature, and they appear capable of significantly affecting CUA results.

## Background

Fundamental to measuring Quality-Adjusted Life Years (QALYs), as used for cost-utility analysis (CUA), are value sets for health states representing health-related quality of life (HRQoL). A value set comprises HRQoL values for all possible health states representable by the health state classification system concerned – of which well-known examples include the EQ-5D [1], HUI [2] and SF-6D [3]. Most systems’ values range between unity for ‘perfect health’ and zero for ‘dead’ , with negative values for states considered worse than dead.

Value sets are created by asking interview or survey participants to value a subset of the system’s health states and then regression models are used to interpolate values for the full set of states. Standard practice is for participants to be drawn from the general population, in their dual roles as tax-payers and potential patients [4-6]. Thus value sets are derived from valuations mostly for hypothetical health states that participants are not personally familiar with and are required to imagine.

A near-universal finding internationally is that patients – i.e. people with direct personal experience of illness or injury – assign different health state valuations than the general population [7,8]. A literature review by De Wit et al. [9] and a meta-analysis by Peeters et al. [10] concluded that patients’ valuations of their own (actual) health states tend to be higher than hypothetical valuations of descriptions of the same states by the general population. In contrast, however, a meta-analysis by Dolders et al. [11], despite finding some evidence for such relationships, found no statistically significant differences overall.

Most studies in the above-mentioned literature pertain to European and North American countries. To our knowledge, no studies comparing patient and general population valuations include data for New Zealand. And yet the EQ-5D system and Devlin et al.’s value set [12], which is based on valuations from a 1999 survey of the New Zealand general population, is widely used for CUA in New Zealand, in particular by the Pharmaceutical Management Agency (PHARMAC) for health technology assessments [6]. It would be useful to know, therefore, whether the above-mentioned international finding that patient valuations tend to exceed general population valuations also applies to New Zealand.

In addition, most previous studies have sampled patients with illnesses such as cancer or stroke rather than injuries (as we do in this paper). It is possible that valuations may differ between people experiencing an illness (often accompanied by an ongoing decline in health prior to clinical diagnosis) and those experiencing an acute-onset change in health status, as caused by injury.

This paper compares Devlin et al.’s general population valuations from the 1999 survey with injured New Zealanders’ valuations of their own health (also represented on the EQ-5D). The latter valuations were collected between 2007 and 2009 in the Prospective Outcomes of Injury Study [13,14]. We also investigate which of the EQ-5D’s dimensions are most strongly associated with the injured population valuations and compare the results with equivalent findings from the 1999 general population study.

## Methods

### Measurement of health state preferences

In both the injured population and general population studies (explained in turn below), health states were represented using the EQ-5D system and participants’ health state preferences were elicited using the visual analogue scale (VAS) method.

Developed by the EuroQol Group, the EQ-5D system represents HRQoL in terms of five dimensions – mobility, self-care, usual activities, pain/discomfort and anxiety/depression – where each dimension has three possible levels of experience: (1) no problems, (2) moderate problems and (3) extreme problems [1]. The three levels combine across the five dimensions to define 243 health states, each representable by a five-digit combination relating to the relevant level within each dimension listed in the order above (e.g. 11111 represents no problems on any dimension). The EQ-5D has been widely used in studies of injury and illness [15,16].

Participants in both studies were asked to indicate on a VAS depicted as a vertical line marked from 0 (“worst imaginable health state”) to 100 (“best imaginable health state”) where they considered the relevant states were for them. Despite lacking the theoretical underpinnings of the standard gamble or time trade-off methods, the VAS has been empirically verified as a valid method for eliciting health state valuations [17,18].

### Injured population study

Injured New Zealanders’ valuations of their own health on the EQ-5D were obtained from the Prospective Outcomes of Injury Study (POIS), a prospective cohort study of injured people aged between 18 and 64 years [13,14]. POIS participants were recruited from entitlement claims registered between December 2007 and June 2009 with the Accident Compensation Corporation, New Zealand’s government-controlled, no-fault injury compensation insurance scheme. All injury types and a wide range of severities were eligible for recruitment to POIS, except for people whose injuries resulted from self-harm or sexual assault.

POIS participants (n = 2856) completed a first interview 3.2 months after injury, with follow-up interviews 4.6 and 12.3 months (medians) after injury. At each interview, participants were asked to represent their own health on the EQ-5D’s five dimensions and then to rate it on a VAS.

### General population study

The general population valuations of EQ-5D health states come from a self-completed postal survey administered to 3000 adult New Zealanders randomly selected from the electoral roll in 1999 [12] (also see [19] and [20]). Three versions of the survey questionnaire were administered, where each version comprised 13 or 14 different health states to be valued on two VAS, with some states repeated across two or more of the versions. Overall, valuations for 23 different health states were collected. For each state, when arriving at their valuation participants were instructed to imagine that it lasts for one year and to disregard what may happen afterwards.

Although 1360 responses were received (a 50% response rate of those who received the survey), 441 were unusable due to: too few health states being valued (because the valuation of so few states undermines their reliability), ‘dead’ being valued as better than full health, or all states being valued the same (considered to be implausible representations of a participant’s preferences). Also, in order to ensure that only data with the strongest claim to validity were analysed, following the approach recommended by Devlin et al. [12], we excluded a further 523 responses that had two or more logical inconsistencies in their valuations, where a ‘logical inconsistency’ occurs when a health state with a less severe problem on a particular dimension than another state, given its problems on the other dimensions are no more severe, is scored *lower* (e.g. state 21111 scored *below* 22111 would be logically inconsistent). The remaining 396 responses represent the highest quality data possible albeit one inconsistency is permitted in order to ensure a reasonable sample size (only 189 responses had no inconsistencies).

In order to calculate health utility values, Devlin et al. rescale each respondent’s valuations to lie on a scale from 0 (representing death) to 1 (representing full health). As hypothetical valuations for death and full health were not collected for POIS participants, we are unable to do a similar rescaling for these data. We therefore use the raw valuations (without rescaling) from the general population study to allow for direct comparisons between the general population and injury population.

### Analyses

Our choice of the appropriate samples from the injured population and general population studies to include in the present analysis was determined primarily by the availability of matching health states for which valuation data were collected – to allow comparison between samples – rather than matching the samples in terms of their socio-demographic characteristics. International evidence has shown that the usual socio-demographic and background variables have no, or at most weak, impact on individual’s health state valuations [11,21,22].

Thus, of the 23 health states for which valuations were collected in the general population study, our protocol was to include only those states which at least two people (in practice, four) from the injured population study reported as their own health state. For each of these health states, an independent samples t-test was used to determine whether the mean valuations from the two studies are statistically significantly different. Robust standard errors were used for each of these tests to allow for the possibility of clustering at the individual level (as each individual could value the same health state at multiple time periods), and critical values were adjusted, using the Holm-Bonferroni correction, to account for the large number of comparisons performed.

In addition, to investigate which of the five EQ-5D dimensions are most strongly associated with the injured population valuations, we estimated equations (1) and (2) below. These two equations are analogous to the equations used to derive Devlin et al.’s two value sets [12], both of which were selected after extensive testing of a wide range of linear and non-linear specifications, following the modelling approach used by Dolan [23].

(1) $$\begin{array}{ll} 100-\mathrm{VA}S_{\mathrm{it}}=\beta_{0} & +\beta_{1}MO_{\mathrm{it}}+\beta_{2}SC_{\mathrm{it}}+\beta_{3}UA_{\mathrm{it}}\\ & +\beta_{4}PD_{\mathrm{it}}+\beta_{5}AD_{\mathrm{it}}+c_{i}+u_{\mathrm{it}} \end{array}$$

(2) $$\begin{array}{ll} 100-\mathrm{VA}S_{\mathrm{it}}=\beta_{0} & +\beta_{1}MO_{\mathrm{it}}+\beta_{2}SC_{\mathrm{it}}+\beta_{3}UA_{\mathrm{it}}\\ & +\beta_{4}PD_{\mathrm{it}}+\beta_{5}AD_{\mathrm{it}}+\beta_{6}N3_{\mathrm{it}}\\ & +c_{i}+u_{\mathrm{it}}, \end{array}$$

where subscript *i* indexes individuals and *t* indexes time periods (interviews).

Both equations were estimated with individual fixed effects. Equation (1) is a linear main effects model in which the effect of changes between levels 1 and 2 and levels 2 and 3 (see Table 1 below) is constrained to being identical. Equation (2) is an extension of equation (1), in which an additional detriment to health is introduced if any of the EQ-5D dimensions are at level 3 (extreme problems). The dummy variables to represent each of these effects are described in Table 1.

**Table 1:** Dummy variables used to model health state valuations

Mobility (MO)	0 if No problems with walking about (level 1)
	1 if Some problems with walking about (level 2)
	2 if Confined to bed (level 3)
Self-care (SC)	0 if No problems with self-care (level 1)
	1 if Some problems washing or dressing self (level 2)
	2 if Unable to wash or dress self (level 3)
Usual activities (UA)	0 if No problems with performing usual activities (e.g. work, study, housework, family or leisure activities) (level 1)
	1 if Some problems with performing usual activities (level 2)
	2 if Unable to perform usual activities (level 3)
Pain/discomfort (PD)	0 if No pain or discomfort (level 1)
	1 if Moderate pain or discomfort (level 2)
	2 if Extreme pain or discomfort (level 3)
Anxiety/depression (AD)	0 if Not anxious or depressed (level 1)
	1 if Moderately anxious or depressed (level 2)
	2 if Extremely anxious or depressed (level 3)
Any dimensions at level 3 (N3)	1 if any dimension is at level 3
	0 otherwise

As explained in Devlin et al. [12], because the EQ-5D system values health states other than full health as negative deviations from 11111 = 1, *VAS*_*it*_ (as explained earlier, rendered on a 0-100 VAS) can be represented as 100 minus the combination of dummy variables and their coefficients (to be estimated). This allowed the dependent variable in equations (1) and (2) to be transformed to 100 - *VAS*_*it*_ (so that higher values correspond to ‘worse’ health states).

Consistent with the explanations above, coefficients *β*_1_-*β*_5_ are interpretable as the decrements to a health state’s valuation of a move from level 1 to 2 or from level 2 to 3 on the respective EQ-5D dimensions, all else being equal. Similarly, coefficient *β*_6_ in equation (2) is interpretable as the extra decrement from any dimension being at level 3 (i.e. additional to the effects captured by *β*_1_-*β*_5_).

### Ethical approval

This study was approved by the New Zealand Health and Disability Multi-Region Ethics Committee (MEC/07/07/093).

## Results

The socio-demographic characteristics of participants in the injured population and general population studies are reported in Table 2. The first column for the injured population study refers to the smaller sample whose valuations were included in the direct comparison of health state valuations (i.e. people who, in at least one of the three interviews, reported a health state that was also included in the general population study), whereas the second column refers to those whose valuations were included in the regression analysis (i.e. those who completed the EQ-5D and VAS at least once in the three interviews).

**Table 2:** Socio-demographic characteristics of participants in the two studies

	**Injured population study**		**General population study**
	**For mean health state values**	**For equations (1) and (2)**	
	**n = 2099**	**n = 2847**	**n = 396**
	**%**	**%**	**%**
*Sex*:			
Female	37.3	38.6	54.3
Male	62.7	61.4	45.0
*Age*:			
18-19 years	3.9	3.6	2.5
20-29	22.1	21.0	14.9
30-39	23.3	22.4	21.9
40-49	21.6	22.8	22.5
50-59	21.6	22.3	17.4
60+	7.5	7.9	20.5
*Ethnicity*:			
European or Pakeha	68.8	67.8	86.5
Māori	18.9	19.8	7.1
Pacific islands^#^	6.6	6.8	1.4
Asian or ‘other’	22.8	22.8	4.1
*Employment: (pre-injury for injured population study)*			
Employed	93.0	91.9	64.1
Retired	0.5	0.7	15.9
Homemaker	1.5	1.4	11.6
Student	2.5	2.3	5.6
Seeking work	0.6	0.9	0.8
Other	1.9	2.8	2.0

^#^Samoan, Cook Island Māori, Tongan and Niuean.

Not all percentages sum to 100 because participants for whom a response was not recorded are not reported here. In the injured population study, participants could report more than one ethnicity.

Of the 2856 participants recruited to the injured population study, 2823 valued their own health on the EQ-5D at the first interview (3-months), 1457 at the second interview (5-months), and 2247 at the third interview (12-months). These three interviews resulted in a total of 6527 valuations, covering 115 of the 243 possible EQ-5D states. Of these 115 states, 18 matched the 23 states included in the general population study, of which 13 were valued by at least two participants, and so they could be included in the present study. Table 3 reports the mean valuations for these 13 states from the two studies.

**Table 3:** Mean health state valuations from the injured population and general population studies

**EQ-5D health state**	**Injured population mean (n)**	**General population mean (n)**	**Difference (SE)**
11111	85.4 (1997)	96.6 (396)	-11.2*** (0.4)
11112	76.9 (152)	73.2 (380)	3.8** (1.4)
11113	72.6 (5)	45.5 (131)	27.1** (8.5)
11121	81.5 (912)	73.6 (390)	7.9*** (0.8)
11122	71.9 (143)	53.8 (112)	18.0*** (2.0)
11131	79.2 (6)	53.0 (137)	26.1* (10.3)
11211	78.7 (110)	78.6 (393)	0.0 (1.7)
12111	74.0 (10)	70.2 (241)	3.8 (4.7)
21111	79.5 (85)	74.3 (388)	5.3** (1.6)
21232	46.2 (21)	32.5 (113)	13.7*** (3.7)
22222	54.9 (158)	41.9 (279)	13.1*** (1.6)
22233	46.3 (4)	22.5 (113)	23.7 (11.2)
22323	31.7 (6)	22.7 (105)	9.0 (7.4)

SE: Standard errors of the estimated mean differences.

*Significant at the 10% level; **Significant at the 5% level; ***Significant at the 1% level.

Significance levels adjusted for multiple comparisons using the Holm-Bonferroni method.

As can be seen in Table 3, the mean health state valuations of the injured population are higher than the hypothetical valuations of the general population for all 13 health states considered except 11111. This difference, which tends to be larger the ‘worse’ the state, is statistically significant at the 10% level for all states except 11211, 12111, 22233 and 22323.

Table 4 presents the estimates of equations (1) and (2) for the injured population study and also, for comparison purposes, the general population study. For both equations, though the five dimensions all have statistically significant effects on participants’ valuations (of the expected positive sign), their relative magnitudes in the respective studies are very different.

**Table 4:** **Estimates of equations (**1**) and (**2**) for the two studies**

	**Equation (1)**		**Equation (2)**	
**Dummy variable**	**Injured population coefficient estimate (SE)**	**General population coefficient estimate (SE)**	**Injured population coefficient estimate (SE)**	**General population coefficient estimate (SE)**
MO	4.06 (0.58)	7.72 (0.34)	4.16 (0.59)	6.62 (0.31)
SC	6.98 (0.66)	5.52 (0.41)	6.82 (0.67)	5.37 (0.37)
UA	5.53 (0.49)	7.06 (0.38)	5.04 (0.53)	3.09 (0.37)
PD	2.40 (0.49)	11.0 (0.29)	2.21 (0.50)	7.94 (0.28)
AD	5.61 (0.58)	11.4 (0.29)	5.37 (0.58)	8.83 (0.28)
N3			2.54 (0.98)	17.7 (0.54)
Constant	16.8 (0.35)	18.6 (0.30)	17.0 (0.36)	18.0 (0.27)
N obs.	6523	5283	6523	5283
N individuals	2847	396	2847	396
R^2^	0.20	0.81	0.20	0.85

SE: Standard errors.

All coefficients are significant at the 1% level.

Consistent with our earlier finding (Table 3) that the injured population own health valuations (means) are mostly higher than the general population hypothetical valuations, almost all of the estimated coefficients for both equations are larger for the general population. For injured people, the most important dimension in terms of negative effects on valuations is self-care (SC), followed by anxiety/depression (AD), usual activities (UA), mobility (MO) and pain/discomfort (PD). For the general population, AD is also an important dimension (most important); however, in contrast to the injured population, SC is the least or second-least important dimension (equation 1 or 2), and PD is the second most important dimension (both equations). The additional decrement to health valuations due to extreme problems on any dimension (N3) is much larger in the general population than in the injured population.

## Discussion

Our finding that injured New Zealanders’ valuations of their own health, represented using the EQ-5D system, are mostly higher than general population valuations of the same (hypothetical) states – with the notable exception of state 11111 (discussed below) – is consistent with the previous international research mentioned in the Introduction.

As discussed by Ubel et al. [24], possible reasons for this phenomenon include: (1) the two groups are, in effect, valuing different health states albeit they are represented identically on the EQ-5D; (2) patients adapt to their poor health states (and so value them relatively high); (3) the ‘focussing illusion’ [25], whereby members of the general population over-emphasise aspects of HRQoL most affected by illness; and (4) a shift of reference points as people assess health states with reference to their current health. See Ubel et al. for discussion of these and other explanations.

The one exception to the finding that injured population valuations exceed general population valuations is the valuation for state 11111: it is *lower* for the injured population than for the general population. Consistent with reason (1) above, perhaps this is because for injured people even when, as represented on the EQ-5D, they are at ‘full health’, to them there are other aspects of their health (not captured by the EQ-5D) that are sub-optimal.

Albeit the differences between the injured population and general population valuations – in the range 3.8 to 27.1 (Table 3) – are statistically significant, how *practically* significant are they? Minimally important differences for the EQ-5D (0-1 scale) have been estimated to be in the range of 0.04 to 0.08 [26-28] – equivalent to 4.0 to 8.0 on a 0-100 scale – which suggests that the above-mentioned differences are also practically significant.

The potential implications of our results for CUA can be illustrated via the example of a CUA of laparoscopic-assisted colectomy (LAC) for colon cancer relative to open colectomy (OC) undertaken by Hayes and Hansen [29]. Applying Devlin et al.’s value set [12] – as explained earlier, derived from the general population study included in the present study – the authors reported two sets of results corresponding to data from two randomised control trials (RCTs): mean QALY gains of 0.018 and 0.049 per patient (depending on the RCT) and costs per QALY of $70,389 and $25,857 respectively.

These results were based on a simple model in which the authors of the CUA assumed that patients were in state 22222 when discharged from hospital and 11111 when they recovered from both LAC and OC (LAC has a quicker recovery time). State 22222 is worth 0.464 in Devlin et al.’s value set. For the demonstration here, it is sufficient to increase 22222’s value by 0.130, the difference between the injured population and general population means for 22222 (see Table 3) after normalising them to the conventional 0-1 scale^a^. The effect of 22222 being worth 0.594 instead of 0.464 is to lower the QALY gains from 0.018 and 0.049 (as above) to 0.013 and 0.037 and to raise the costs per QALY from $70,389 and $25,857 to $94,661 and $34,422 respectively. These revised cost-per-QALY estimates are approximately one-third larger than the original estimates. Although cost-effectiveness acceptability thresholds for New Zealand are not reported, such relatively large increases could, conceivably, result in LAC going from being cost-effective (if general population valuations were used) to being not cost-effective (if patient valuations were used). Note, though, the calculations above are intended only to illustrate the possible effects of differences between the injured population and general population valuations, not to serve as a bona fide CUA.

### Strengths and weaknesses of the study

The main strength of this study is that, to the best of our knowledge, it is the first comparison of injured people’s valuations of their own health, represented using the EQ-5D system, vis-à-vis general population valuations of the same EQ-5D states for New Zealand.

This study has several potential weaknesses. As discussed in the Methods section, it was not possible to ensure that the samples employed from the general and injured populations were similar in terms of their socio-demographic characteristics; participants in the injured population study were younger, more likely to be male, employed and of Māori, Pacific Islands, Asian or ‘other’ ethnicity (not European or Pakeha). However, as also discussed earlier, international evidence suggests that this should not have a significant effect on health state valuations [11,21,22]. Another potential weakness is that the two sets of valuations are not from the same data-gathering exercise. Instead, they were run independently, separated in time by almost a decade (i.e. 1999 versus 2007-09). New Zealanders’ health state preferences could, potentially, have changed over this time period; however, the literature provides no indication as to whether this is likely to be a serious problem. Also, the general population study used a self-completed postal questionnaire whereas the injured population study, as part of the Prospective Outcomes of Injury Study, was mostly interviewer-administered. These differences between data collection methods have the potential to cause information bias; again, we cannot determine the nature or possible extent of this potential bias.

## Conclusions

Consistent with the international literature, we find that injured people’s VAS valuations of their own health, represented using the EQ-5D system, are mostly higher than the general population’s hypothetical valuations of the same EQ-5D states for New Zealand. These differences are practically significant in the sense that they are larger than minimally important differences for the EQ-5D from the literature, and they appear capable of significantly affecting CUA results; hence they could potentially influence health technology assessments.

Our results are further evidence that it can make a difference whose health state valuations are included when value sets are being created. However, this does not mean, necessarily, that patients’ (or injured people’s) valuations *should* be used instead of the general population’s. That is a whole other matter that ultimately depends on the decision-making context and the purpose of the analysis [8]. The consensus seems to be that for decisions at the individual-patient level, such as treatment options, the patient’s own valuations should be used. For decisions at the aggregate level, such as health technology assessments, value sets representing the general population should be used.

## Endnote

^a^Note that Devlin et al.’s value of 0.464 is not directly comparable with the general population value in Table 3 because Devlin et al.’s value set comes from estimating equation (2) above rather than it being a mean value, and also because, as is conventional when estimating values, the underlying survey valuation data were ‘rescaled’ relative to each participant’s hypothetical valuations of ‘dead’ and ‘perfect health’ (which were not collected in the injured population study).

## Abbreviations

AD: Anxiety/depression dimension on the EQ-5D; CUA: Cost-utility analysis; HRQoL: Health-related quality of life; LAC: Laparoscopic-assisted colectomy; MO: Mobility on the EQ-5D; N3: Extreme problems on any EQ-5D dimension; OC: Open colectomy; PD: Pain/discomfort on the EQ-5D; PHARMAC: Pharmaceutical Management Agency; POIS: Prospective Outcomes of Injury Study; QALYs: Quality-Adjusted Life Years; RCT: Randomised control trial; UA: Usual activities on the EQ-5D; VAS: Visual analogue scale.

## Competing interests

All authors declare that they have no financial interests that may be relevant to the submitted work. SD is a member of the EuroQol Scientific Committee, which developed the EQ-5D instrument used in the study.

## Authors’ contributions

RW led the preparation of this article, with guidance from PH and SD, and conducted the statistical analysis. SD leads the Prospective Outcomes of Injury Study (POIS) research team. PH and JL are POIS co-investigators. All authors contributed to the writing of the final manuscript and approved it.
